# Non-analgesic effects of opioids: Topical application of Eucerin-based ointment containing opium on the healing process of thermal skin damage in rats

**DOI:** 10.1371/journal.pone.0313659

**Published:** 2024-11-22

**Authors:** Omid Mehrpour, Khadijeh Farrokhfall, Kobra Naseri, Samaneh Nakhaee

**Affiliations:** 1 Michigan Poison & Drug Information Center, School of Medicine, Wayne State University, Detroit, Michigan, United States of America; 2 Medical Toxicology and Drug Abuse Research Center (MTDRC), Birjand University of Medical Sciences (BUMS), Birjand, Iran; 3 Pharmaceutical Sciences Research Center, Birjand University of Medical Sciences, Birjand, Iran; Qassim University, SAUDI ARABIA

## Abstract

The present study aimed to investigate the efficacy of different doses of Eucerin-based ointment containing opium compared to routine treatment on experimentally induced burn injury. Male Wistar rats were divided into five experimental groups for topical application: silver sulfadiazine 1% (SSD), Eucerin-based ointment containing opium at concentrations of 0.05%, 0.5%, and 5%, and a Eucerin-based ointment containing 0.05% opium plus SSD (mixed group), following burn wound induction using the comb burn model. An equal volume of different ointments was administered topically. The wound appearances of different groups were photographed at different time points for 21 days. In addition, blood and skin samples were taken 2 and 10 days after thermal injury to assess inflammatory parameters and oxidative stress markers. Also, the liver enzyme activity and kidney function tests were assessed on day 10. The body weight in Opium 5% significantly decreased compared to all other groups after ten days. The wound area was significantly reduced (P<0.05) in three groups: Opium 0.05%, Opium 0.5%, and mixed, compared to the SSD group on days 14 and 21. On day 21, the wound size in the Opium 5% group was significantly larger than that in the SSD group. Significantly lower serum levels of MDA were observed in all groups compared to the SSD group on days 2 and 10. There were no significant differences between treatment groups for concentrations of TNF-α in serum and burned skin samples (p>0.05). The serum concentrations of interleukin-10 in the mixed group were significantly higher compared to the SSD group on day 10. After ten days, groups were not different regarding AST, ALT, and creatinine concentrations (p>0.05). Histopathological analysis revealed that the wound healing efficacy of opium at its lowest concentration (0.05%) surpassed that of silver sulfadiazine (SSD). Furthermore, the combination of 0.05% opium with SSD enhanced the wound repair potential of SSD in burn injuries. This study showed that opium ointment delayed wound closure dose-dependently. Low doses of topical opium ointment and its mixed application with silver sulfadiazine had a protective role in thermal-induced injury.

## Introduction

Burn injuries, depending on their type and severity, can affect various bodily systems and are among the most painful [1]. In addition, many internal and external factors affect burn healing [2]. For example, perfusion, inflammation, and oxidative balance changes may be involved in wound healing and the extent of the necrotic area [3]. Despite significant advancements in burn injury management and treatment, there remains a need for more effective therapeutic agents [1]. Due to its antimicrobial properties, routine treatment of burn wounds has centered on dressings containing silver sulfadiazine (SSD) as a gold standard for decades [4]. However, receiving widespread acceptance, it possesses various disadvantages, such as limited penetration in lesions, low solubility, adhesive pseudo-scar formation, delay in the development of granular tissue, risk of silver toxicity, and neutropenia [4–6].

Opioids are commonly used for pain control in burn injuries, both in the initial stage and during painful procedures like dressing changes and wound debridement [7]. Increasing numbers of evidence suggest that opioids and opioid receptors expressed in the skin have strong analgesic properties and are effective in wound healing [8, 9]. Opioid receptors can influence wound healing mechanisms and intercellular adhesion [10]. Some studies suggest the significant role of different opioid receptors, including mu, delta, and kappa receptors, in skin proliferation, migration, and differentiation factors essential for wound healing [8–11].

Additionally, substances with opiate activity, such as morphine, have been reported to enhance the wound-healing process. At the same time, opioid antagonists such as Naloxone suppress wound healing and prevent the healing process caused by opiate peptides [12]. Opioids can alter the healing process probably via altering the inflammatory milieu of the wounds [2]. Opioid receptors are detected in lymphocytes, macrophages, monocytes, and granulocytes. Leukocytic opioid receptors have biochemical and pharmacological properties similar to neuronal opioid receptors. They are shown to modulate *in vitro* the proliferation of immune cells, expression of cytokine and chemokine receptors, and synthesis and secretion of cytokines. However, these effects were contradictory in some cases [13]. Additionally, the migratory ability of human keratinocytes plays a crucial role in the re-epithelialization stage of wound healing [11], and previous reports have shown that opioid agonists can stimulate the migration of human keratinocytes [11].

The typical side effects induced by opioids may limit their systemic application. Therefore, their topical application has been investigated as an alternative in skin wounds [13]. The application of opium, a natural opioid, on wound burns is required to be formulated as a topical therapy. Eucerin, a purified product containing lanolin alcohol, is a widely used emulsifier in topical formulations. It was created as a water-soluble foundation for medicinal salves. Lanolin alcohol enables the combination of lipids with water to form a consistent emulsion. The substance contained significant cholesterol and was recognized as an excellent ingredient for skin care cosmetics. It has also been used as a skin emollient for thousands of years and is likely to promote wound healing [14–17].

However, there is research on opioids and Eucerin on wound healing. Therefore, to gain a better understanding of their role in burn wound healing, we conducted an experimental study to assess the effects of different doses of Eucerin-based ointment containing opium compared to routine treatment on the burn healing process.

## Material and methods

### Experimental protocol

This experimental study assessed the efficacy of a Eucerin-based ointment containing opium versus standard treatment for burn healing. The study used sixty-five male Wistar rats weighing 200–270 g, obtained from the Research Centre of Experimental Medicine at Birjand University of Medical Sciences. The rats were housed under controlled conditions with a temperature of 22±2 °C and a 12-hour light/dark cycle and were given free access to water and a standard diet. The study adhered to ethical guidelines and was approved by the Research Ethics Committee of the National Institute for Medical Research Development (NIMAD) (IR.NIMAD.REC.1397.262). The study was designed to minimize the number of animals required, all procedures were performed under ketamine and xylazine anesthesia, and all efforts were made to minimize suffering. This study is reported based on the ARRIVE guidelines.

For the experiment, anesthesia was administered to the rats via intraperitoneal injection of ketamine (80 mg/kg) and xylazine (20 mg/kg). The back area of each rat was shaved with an electric hair clipper. After animals reached the appropriate depth of anesthesia, the thermal injury was created using the comb burn model [18]. In this model, a brass comb with four prongs is heated in boiling water until it reaches a stable temperature. The resulting burns on an animal are four in number and separated by three skin areas that are not damaged. The progression of the burns horizontally can be observed by monitoring these undamaged areas over time. A brass comb was heated in boiling water for 3 minutes. It was then applied vertically on the skin of the dorsal area without pressure for 20 seconds, resulting in a second- degree four rectangular burned areas of 20 mm^2^ separated by three unburned spaces (ischemic or stasis areas) (Fig 2a). Both sides of the dorsal area were burned, and no dressing was applied. All animals received 1 ml/kg normal saline 0.9% intraperitoneally after thermal injury. Following the induction of burn wounds, the animals were randomly allocated into five experimental groups, each comprising 13 rats:

1. Silver Sulfadiazine Group (SSD): This group received a topical application of 1% silver sulfadiazine ointment (*Iran Najo Pharmaceutical Co*, Iran), each 100 g containing 1 g of silver sulfadiazine.
2. Opium 0.05% Group (OP 0.05%): Rats in this group were treated with a topical application of a Eucerin-based ointment containing 0.05% opium.
3. Opium 0.5% Group (OP 0.5%): Rats in this group were treated with a topical application of a Eucerin-based ointment containing 0.5% opium.
4. Opium 5% Group (OP 5%): Rats in this group were treated with a topical application of a Eucerin-based ointment containing 5% opium.
5. Mixed Group: This group received a topical application of a Eucerin-based ointment containing 0.05% opium plus SSD.

**Fig 1:**
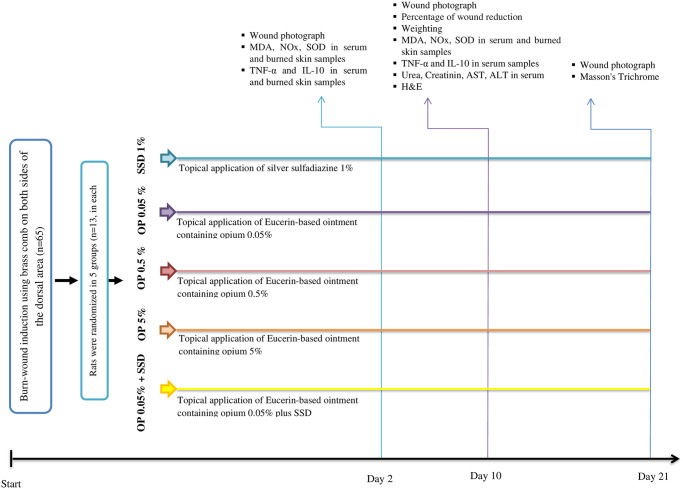
Flow diagram of different experiments and the study process.

Subsequently, each group was further divided into three subgroups based on the treatment duration: 2 days, 10 days, and 21 days. The treatments were initiated one hour after burn-wound induction and administered daily for the respective durations. The experimental design and the study process are illustrated in Fig 1.

The preparation of the 5% (w/w) opium ointment involved mixing 5 g of opium powder (EN 100-016/96, *Faran-Shimi* Pharmaceutical Company, Tehran, Iran) with 95 g of Eucerin ointment (Batch No: 210504, *Sepidaj Pharmaceutical Company*, Iran). An equal volume of the various ointments was applied topically across the different groups.

### Measurements and outcomes

Before the burn induction and ten days following treatment, the weight of each animal was recorded. Wound photographs for each group were taken on days 0, 2, 10, 14, and 21 post-burn injury and analyzed with *ImageJ* software to measure the wound area. The wound size reduction was calculated using the formula:

$$\text{Percentage}\;\text{of}\;\text{wound}\;\text{area}=\left[\left(\text{wound}\;\text{area}\;\text{on}\;\text{day}\;0-\text{wound}\;\text{area}\;\text{on}\;\text{the}\;{\text{X}}^{\text{th}}\;\text{day}\right)/\text{wound}\;\text{area}\;\text{on}\;\text{day}\;0\right)]\times 100.$$

Animals were sacrificed under deep anesthesia (ketamine/xylazine 80:20 mg/kg, IP) and blood and skin samples were collected for further assessment. Blood samples were taken through cardiac puncture on days 2 and 10 post-injury, followed by centrifugation of the coagulated blood samples for 10 minutes at 3000 rpm. The serum was then separated and stored at -20°C for biochemical evaluation. Full-thickness burned skin samples were excised and divided into two parts: one part was stored for histopatological examination, and the other was stored at -20°C. One part of the excised skin was weighed and homogenized (MICCRA-D1, Germany) in cold phosphate buffer saline (PBS) (pH = 7.4). The homogenate mixture was centrifuged (10000 rpm, 20 min at 4 °C), and the supernatant was collected and stored to determine inflammatory and oxidative stress parameters at -20 °C. The serum and skin inflammatory parameters, including tumor necrosis factor-α (TNF-α) and interleukin-10 (IL-10), were evaluated using enzyme-linked immunosorbent assay (ELISA) kits (ZellBio GmbH, Ulm, Germany. cat.no. ZB-10764C-R9648 and ZB-10108C-R9648, respectively) according to the manufacturer’s instructions. Additionally, oxidative stress markers, such as thiobarbituric acid reactive substances (TBARS) for MDA measurement, superoxide dismutase (SOD), and nitrite/nitrate (NOx), were measured in the serum and burned skin samples. Nitrite/nitrate (NOx) levels were assessed using the Griess reaction as previously described [19–21], while TBARS levels for MDA measurement were determined using Uchiyama and Mihara’s method [22]. SOD activity was measured using Nasdox^™^–Superoxide Dismutase Non-Enzymatic kit (Code: NS-15033, *Navand Salamat*, Iran). Different parameters were expressed per milligram of protein in homogenized tissues using a commercial protein assay kit (Code: NS-15074, *Navand Salamat*, Iran). The liver enzyme activity (alanine transaminase (ALT) and aspartate transaminase (AST)) and kidney function tests (Creatinine (Cr) and urea) were determined using Roche diagnostic kits (Germany) and auto analyzer machine (Cobas 6000, Roche, Germany). The C501 module, the Cobas 6000 (Roche, Germany), is a biochemical analyzer using spectrophotometric measuring (photometric detector) for assay of the above liver and function tests [23, 24].

### Histopathological assessments

In order to evaluate the healing process of wounds in the different studied groups, skin biopsies of the wounded area were taken for histopathological assessment on the 10^th^ and 21^st^ post-burn days. The skin samples were fixed in 4% paraformaldehyde solution and then processed for paraffin embedding by routine histological procedures, and 5μm thickness sections were prepared. The slides were stained with hematoxylin and eosin (day 10) and Masson’s trichrome (day 21) and analyzed under a light microscope (Euromex-CMEX-10). Since the cutaneous wound healing process involves both the epidermis and dermis, we used a systematic evaluation scoring system in which epithelialization and healing of the dermis are scored [25]. Briefly, a 0–7 scoring system was used to assess the healing of the epidermis based on the condition of the wound crust and epithelialization state (Table 1) [25]. Similarly, the dermis healing process was scored (0–7) based on the semi-quantitative evaluation of adipose cells, inflammatory cells, fibroblasts, the state of collagen deposition, and the formation of hair follicles (Table 2) [25].

**Table 1:** Histological scoring system of burned epidermis.

Score	Crust status	Epithelialization	Rete ridge
0	Loosely attached	no	-
1	Tightly attached	Minimal	-
2	Tightly attached	Mild	-
3	Tightly attached	Moderate	-
4	No crust attached	Moderate	-
5	No crust attached	Severe	-
6	No crust attached	Complete	-
7	No crust attached	Complete	+

**Table 2:** Histological scoring system of burned dermis.

score	Adipose cell	Inflammatory cell	Fibroblast	Collagen deposition	Hair follicle
0	+++	+	-	-	-
1	++	++	+	-	-
2	+	++	+	-	-
3	+	+++	+	-	-
4	+	+++	++	+	-
5	-	++	+++	++	-
6	-	+	+++	++	-
7	-	+	++	+++	+

https://www.ncbi.nlm.nih.gov/pmc/articles/PMC7675209/table/tbl02/?report=objectonly

-: absent; +: mild; ++: moderate; +++: severe

### Statistical analysis

The data collected were analyzed using the SPSS software (Statistical Package for Social Sciences, Microsoft, v. 22). One-way analysis of variance (ANOVA) was used to compare the biochemical data between the experimental groups, followed by Tukey’s post hoc test. A P-value below 0.05 was considered significant.

## Results

### The macroscopic appearance and wound size reduction

**Fig 2:**
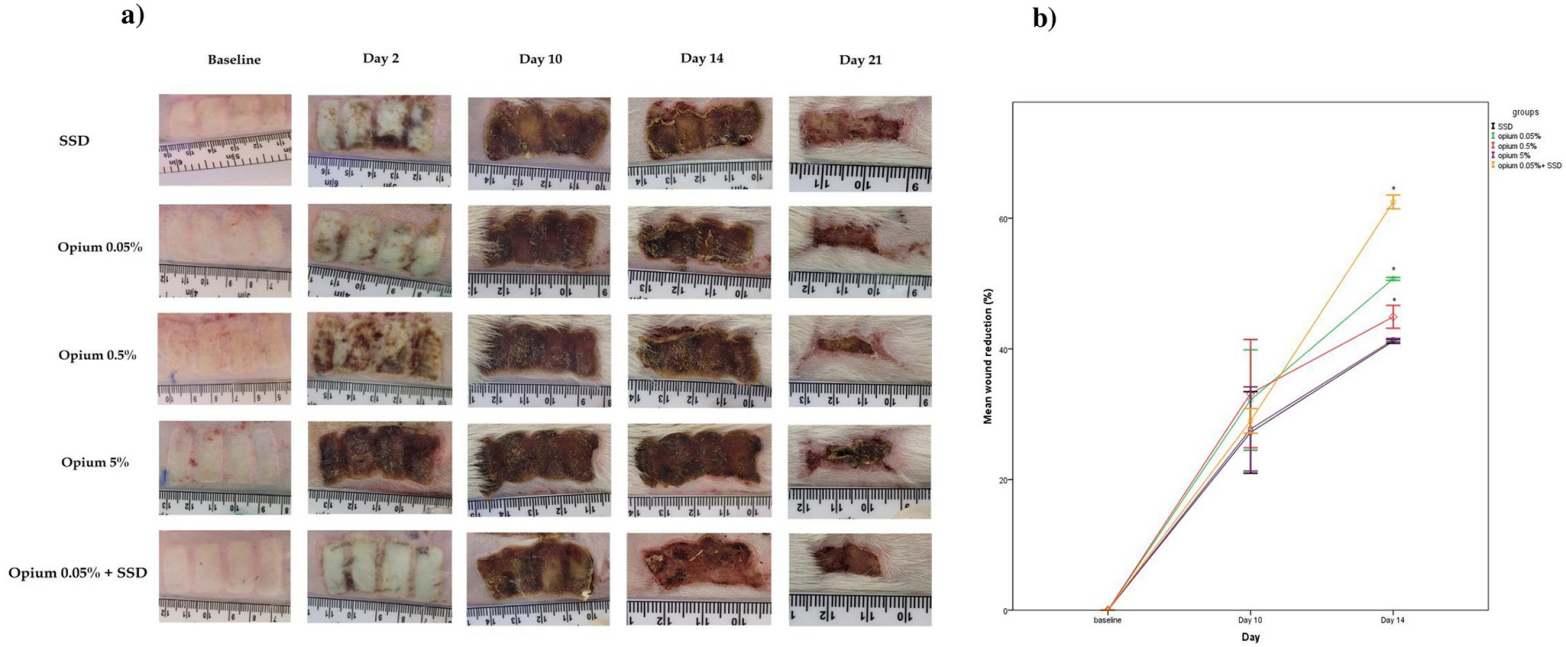
The wound appearance of different groups during the period of study (a) and the percentage of wound reduction in treated groups on different days (b). *significant as P < 0.05 compared to the other groups.

The wound’s macroscopic appearance in different groups has been shown in Fig 2a. The healing process was accelerated during 21 treatment days in the groups of Opium 0.05%, opium 0.5%, and mixed compared to other groups. As shown in Fig 2b, the mean percentage of wound size reduction had no significant differences between groups on day 10, but this variable significantly differed between groups on day 14. The results showed that wound surface was significantly reduced (P<0.05) in three groups of Opium 0.05% (50.72% ± 0.21), Opium 0.5% (44.9% ± 1.75), and a mixed group (62.49% ± 1.04) compared to the SSD group on day 14 (41.16%± 0.3). The wound size of the SSD and opium 5% groups was not significantly different on day 14.

### The effects on oxidative stress markers including MDA, SOD, and Nitric Oxide Metabolites (NOx) in serum

**Fig 3:**
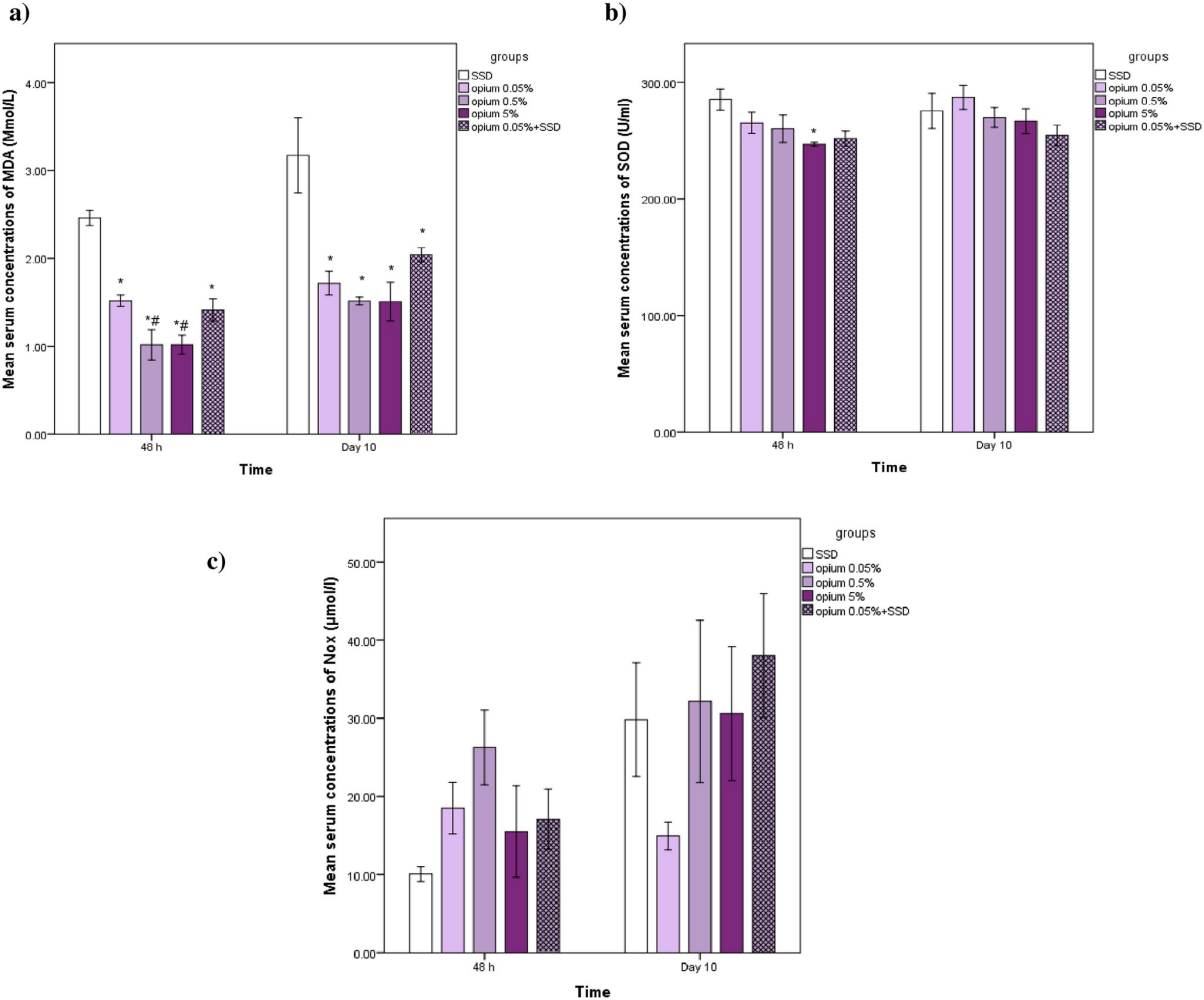
Serum concentrations of malondialdehyde (a) and super oxide dismutase (b) and NOx (nitrate +nitrite) (c) in different experimental groups on day 2 and 10. n = 6 or 5 in each group. Data has been shown as mean± SD. *: compared to SSD group, #: compared to opium 0.05%.

As shown in Fig 3, significantly lower serum levels of MDA were observed in all groups compared to the SSD group on days 2 and 10 (p<0.05) (Fig 3a). The results showed that SOD activity in Opium 5% was significantly lower than the SSD group on day 2 (Fig 3b). The nitrosative stress was measured by nitrite + nitrate content (NOx). No significant differences were found between treatment groups for serum Nox concentrations on days 2 and 10 (p<0.05) (Fig 3c).

### The effects on oxidative stress markers, including MDA, SOD, and Nitric Oxide Metabolites (NOx) in the burned skin samples

**Fig 4:**
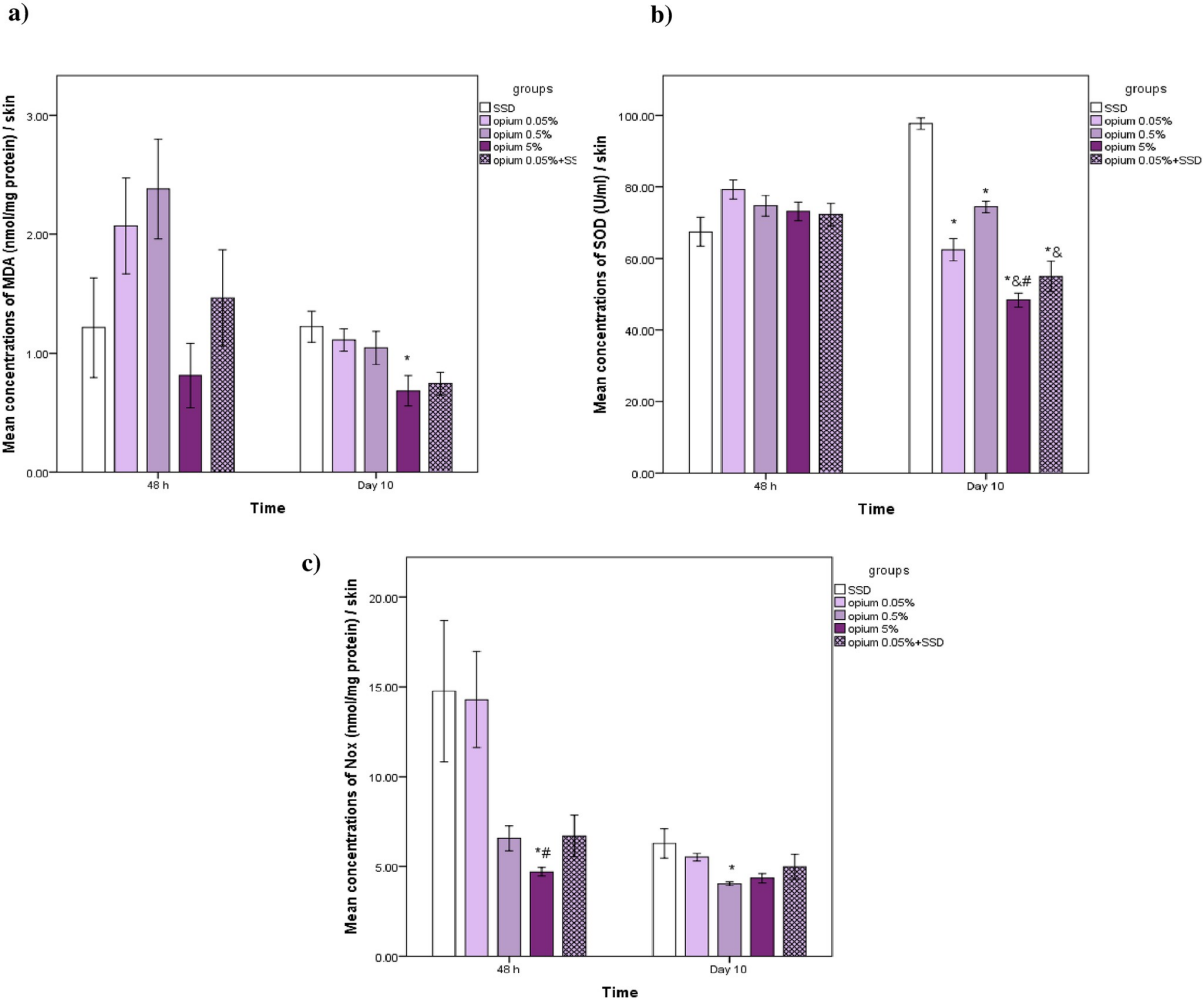
The concentrations of malondialdehyde (a), super oxide dismutase (b), and NOx (nitrate +nitrite)(c) in the burned skin samples of different experimental groups on day 2 and 10. n = 6 or 5 in each group. Data has been shown as mean± SD. *: compared to SSD group, #: compared to opium 0.05%, &: compared to opium 0.5%.

The mean concentrations of MDA in the burned skin samples of different experimental groups showed significantly lower levels in the Opium 5% group compared to the SSD group on day 10 (p<0.05) (Fig 4a). All groups showed significantly lower levels of SOD activity compared to the SSD group on day 10 (Fig 4b). Also, in the opium 5% group, lower levels of SOD activity were observed compared to two other groups of opium 0.5% and Opium 0.05%. Compared to the SSD group, the mean concentrations of NOx on the second day were significantly lower in the burned skin samples of Opium 5%. On the tenth day, Opium 0.5% had significantly lower NOx concentrations. (p<0.05) (Fig 4c).

### The effects on inflammatory parameters (TNF-α and IL-10) in serum and burned skin samples

**Fig 5:**
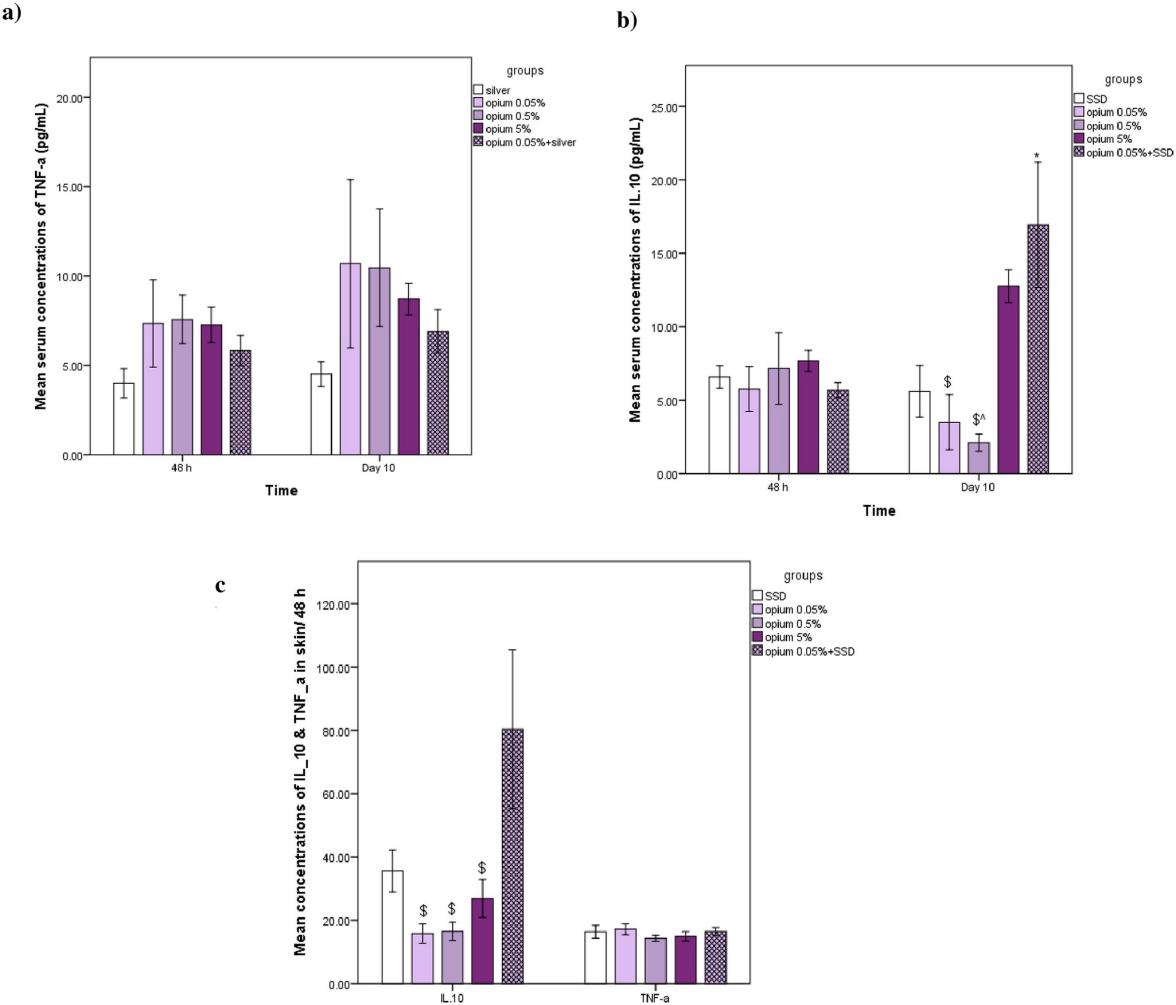
The concentration of inflammatory parameters (TNF-α and IL-10) in serum (a and b) and burned skin samples (c) of different experimental groups on day 2 and 10. n = 6 or 5 in each group. Data has been shown as mean± SD. *: compared to SSD group, ^: compared to opium 5%, ^$^: compared to mixed group.

There were no significant differences between treatment groups for concentrations of TNF-α in serum and burned skin samples (p>0.05) (Fig 5a and 5c). The results showed that the serum concentrations of interleukin-10 in the mixed group were significantly higher compared to the SSD group on day 10 (p<0.05) (Fig 5b). Also, higher concentrations of IL-10 were observed in the mixed group compared to Opium 0.5% and 0.05% in both serum on day ten and skin samples on day 2 (p<0.05) (Fig 5b and 5c).

### The effects on body weight, liver, and kidney functions

**Fig 6:**
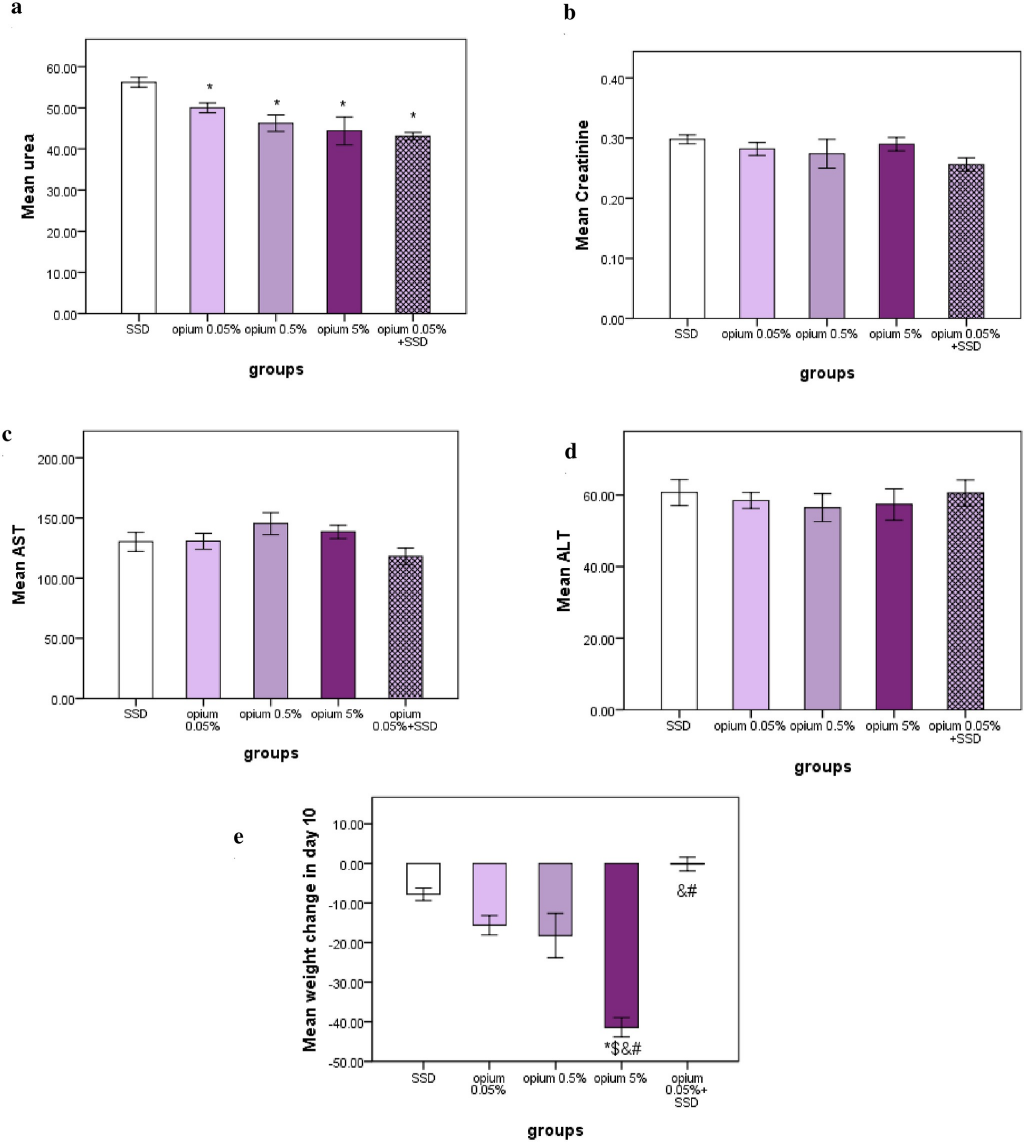
Renal (urea and creatinine) (a, b) and Liver function (ALT and AST) (c, d) test in serums of different experimental groups and body weight changes (e) on day 10. n = 6 or 5 in each group. Data has been shown as mean ± SD. *: compared to SSD group, #: compared to opium 0.05%, &: compared to opium 0.5%, $: compared to mix group.

The body weight in Opium 5% significantly decreased compared to all other groups after ten days (Fig 6e). Also, Opium 5%, 0.5%, and 0.05% had significantly more weight reduction in comparison with the mixed group (p<0.05) (Fig 6e). The mean urea serum levels were significantly higher in the SSD group compared to other groups (p<0.05) (Fig 6a). However, there were no significant differences between experimental groups in terms of AST, ALT, and Creatinine concentrations after ten days (p>0.05) (Fig 6b–6d).

### Histopathological findings

**Fig 7:**
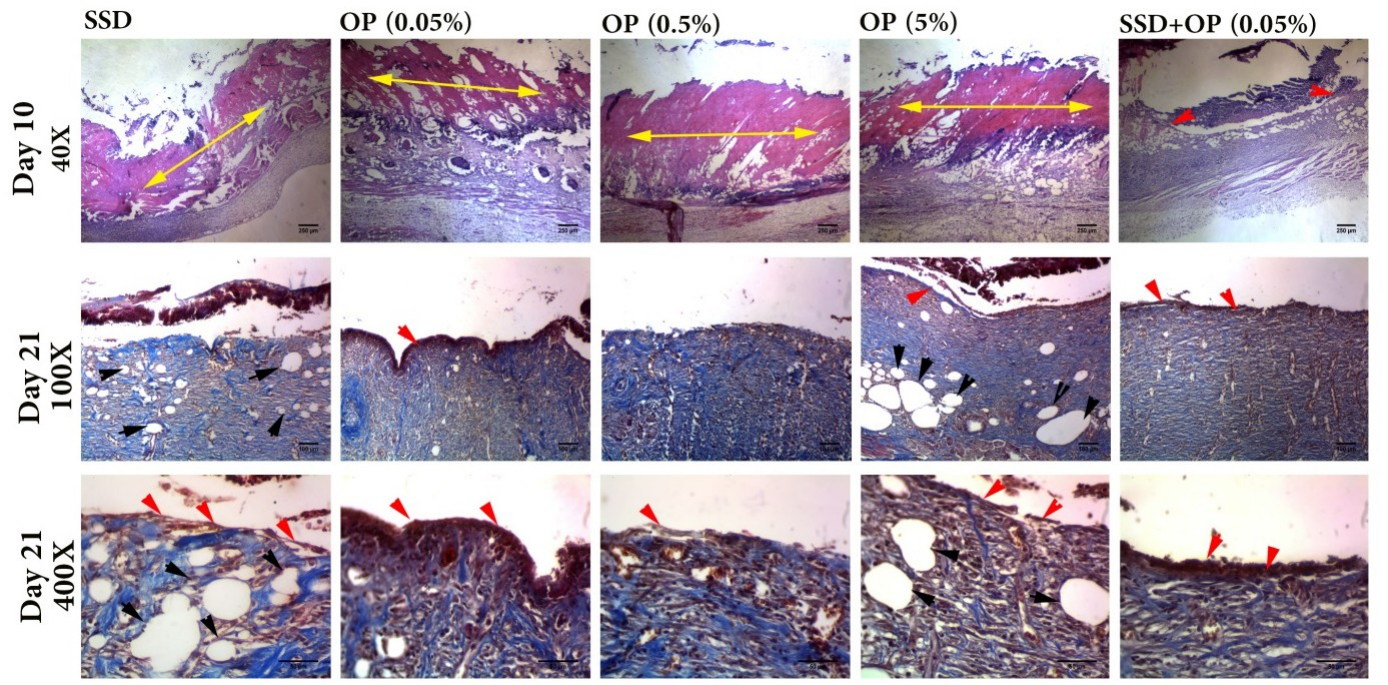
The representative histological images of rat deep partial-thickness burns after 10 and 21 days of treatment with silver sulfadiazine 1% (SSD), opium 0.05% (OP0.05%), opium 0.5% (OP0.5%), opium 5% (OP5%), and SSD plus opium 0.05% (SSD+OP0.05%). The slides were stained with hematoxylin and eosin (day 10) and Masson’s trichrome (day 21) with 40X (scale bar = 250μm), 100X (scale bar = 100μM) and 400X (scale bar = 50μM). Double headed arrows indicated burn crust; red arrow heads indicate epithelializarion and black arrow heads indicate adipocytes. Collagen fiber bundles stained in blue (day 21).

**Fig 8:**
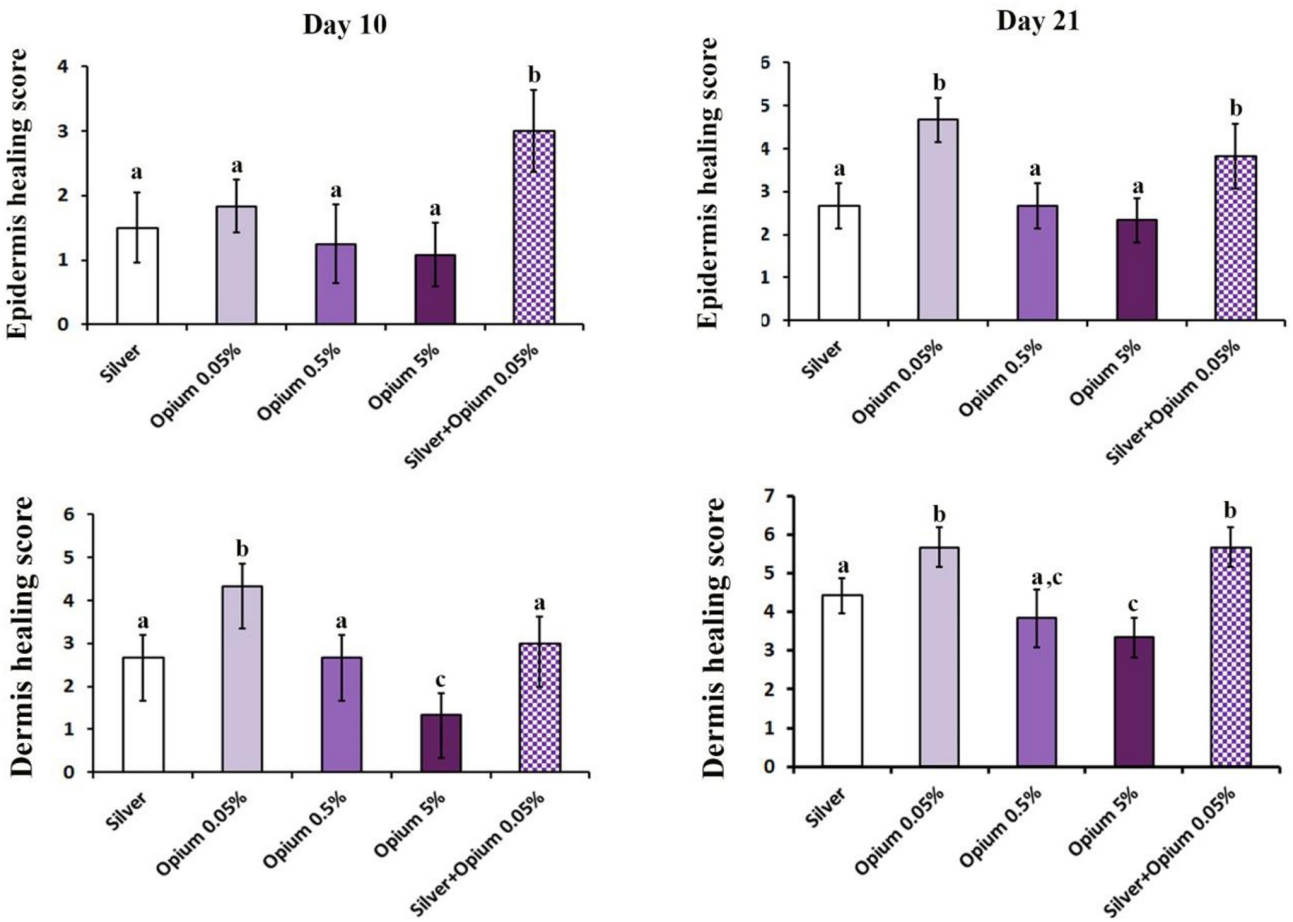
The mean scores of epidermis and dermis healing processes in burned rats after 10 and 21 days treatment with silver sulfadiazine 1% (silver), opium 0.05% (OP 0.05%), opium 0.5% (OP 0.5%), opium 5% (OP 5%), and silver plus opium 0.05% (silver + OP 0.05). Each value represents the mean ± S.D. Differences among groups were analyzed by a one-way analysis of variance (ANOVA) followed by Tukey’s post hoc test. Different letters (a-c) show a significant difference (*p* < 0.05), and the same letters indicate a non-significant difference between groups.

By day ten after the induction of the burn injury, histopathological features of the burned skins showed full dermis damage, representing the formation of deep partial-thickness second-degree burn (Fig 7). In the burn surfaces of the SSD, OP 0.05%, OP 0.5%, and OP 5% groups, the wound crust was attached, but as it was loosely attached in the SSD+OP 0.05% group (as observed in Fig 2a), resulting in its disappearance during the histopathological process. In all the studied groups, the entire epidermis was absent, or only minimal epithelialization on the edges of the burn area was observed (Fig 7, red arrowheads). There was no significant difference in the epidermis healing score between the SSD, OP 0.05%, OP 0.5%, and OP 5% groups (p > 0.05 for all comparisons). However, the score of the group treated with SSD and OP 0.05% was significantly higher than all other groups (p < 0.05 for all comparisons), according to data in Fig 8. The highest dermis healing stage was observed in the OP 0.05% group, whereas the lowest was in the OP 5% group (Fig 8). The other studied groups had no significant difference in the dermis healing stage. In the OP 0.05% group, the proportion of inflammatory cells and fibroblast cells was prominent, while in the SSD, OP 0.5%, and SSD+OP 0.05% groups, inflammatory cells were prominent. Adipocytes and inflammatory cells were abundant in the OP5% group (Fig 7).

Histopathologically, after 21 days of wound burn injury treatment, the highest epidermis healing was observed in the OP 0.05% and SSD+OP 0.05% groups representing moderate epithelialization (2–3 epithelial cell layers), which were significantly higher than the other studied groups (p<0.05 all) (Figs 7 and 8). There was no significant difference in epidermis healing score between OP 0.05% and SSD+OP 0.05% (4.5±1.04 vs. 4.00± 0.63, p = 0.73). Evaluation of the dermis healing process showed that the process was accelerated in the OP 0.05% and SSD+OP 0.05% groups compared to SSD, OP 0.5%, and OP 5% groups. In the dermis of OP 0.05% and SSD+OP 0.05% groups, the score of collagen deposition and fibroblast cells proportion were higher than the SSD, OP 0.5%, and OP 5% groups (Fig 7). Overall, the wound healing activity of opium at the lowest concentration (0.05%) was superior to SSD, and its combination with the SSD increased the SSD burn wound repair potential.

## Discussion

In photograph and pathological assessments, 21 days after thermal injury, opium ointment delayed wound closure dose-dependently, and high-dose opium application increased residual scar tissue on burned skin. Also, we observed a significant reduction in wound size of groups treated with Opium 0.05%, 0.5%, and mixed ointment compared to the SSD group. Previous investigations have assessed the effect of various opioids on the healing process of different types of wounds, with both positive and negative results.

An investigation examined the wound-healing effects of topically applied opioids (morphine, hydromorphone, and fentanyl) in rats. Topical opioids improved wound closure in a -reversible manner with Naloxone, especially during the first four days when no improvement was observed in control wounds [26]. In a different experimental investigation, the opioid peptide Dalargin stimulated the growth of capillaries and fibroblast proliferation using intraperitoneal administration and local application. In addition, it accelerated the maturation of granulation tissue and epithelialization and reduced the process of wound healing in rats [27]. To clarify the pathophysiological effects of the delta-opioid receptor (DOR) system in the skin, Bigliardi et al. (2006) compared wound healing in DOR knock-out (KO) and wild-type (WT) mice. In the intact skin of KO mice, there was a changed differentiation pattern and phenotype of a thinner epidermis. Also, observations on wounded skin showed that DOR deletion could result in delayed wound healing and hypertrophic epidermis [9]. Bigliardi et al., in another study, examined the pattern of human keratinocyte migration after exposure to various agonists and antagonists of the mu-opiate receptors. They suggest that mu-opiate receptor agonists can stimulate cultured keratinocyte migration. These effects show that the opioid peptides released in wounds can play an important role in tissue remodeling and re-epithelialization [11]. Analgesic opioids and endorphins have been hypothesized to stimulate angiogenesis, endothelial proliferation, and wound survival via mitogen-activated protein kinase/extracellular signal-regulated kinase (MAPK/ERK) signaling [8]. Opioids can also regulate vascular physiology. They may improve the healing process by increasing perfusion in and around the wounds. Also, they can stimulate revascularization and reduce neuro-inflammation in the wounds by suppressing substance P release both centrally and peripherally, which may contribute to opioid-induced healing [8].

A delay in wound healing caused by opioids has been characterized, especially in the early stages, mostly in the inflammatory phase of healing or in high-dose opioids. Lam et al. (2008) demonstrated impaired angiogenesis and mobility of endothelial progenitor cells following long-term use of high-dose morphine in mice [28]. Rook et al. (2008) reported that topical morphine gel (IntraSite Gel containing 5 mM morphine) caused delayed wound closure in a dose-dependent pattern in rats on days 0–3 post-wound induction. Indeed, a decrease in the thickness of the skin and an increase in residual scar were reported in the morphine-treated group, which was explained by a decrease in the number of active myofibroblasts. However, the outcome showed no differences between the control and opioid-treated animals [29]. Based on these results, the initial delay of wound closure was transient and followed by an accelerated re-epithelialization. Those findings are supported by *in vitro* studies highlighting the opioid effects on re-epithelialization and keratinocyte migration [11, 30]. The report of evidence on wound closure delays with topical morphine application can be due to alterations in the initiation and early processes of wound healing. So, the onset of inflammation delay can interrupt the timing of subsequent events [29].

Conflicting reports exist regarding the impact of oral opium addiction on wound healing [2, 7, 31]. Individuals with opioid dependence or who use opioids for therapeutic purposes, such as cancer pain management, frequently face issues with delayed wound healing. Chronic opioid usage may hinder innate immune responses, impacting subsequent immune processes. The underlying mechanisms associated with this phenomenon remain unclear [13]. Aside from the immunosuppressive effects that opioids may have, other factors can contribute to impaired wound healing in individuals with opioid dependence. Malnutrition and weakened immune systems are common in drug dependents and individuals with cancer, negatively impacting wound healing.

Another result of the current study is that all doses of opium ointment reduced lipid peroxidation and oxidative stress in days 2 and 10 post-burn induction. However, the effective findings of high-dose opium on lipid peroxidation were not correlated with the photographic and histological evaluation findings. A wide range of experimental studies has shown opium consumption can lead to oxidative stress, namely an increase in MDA level and antioxidant enzymes decrement [32–37] that it participates in opium addiction and dependency [38]. Also, opium consumption/addiction is associated with oxidative stress [39–41]. However, other studies introduced morphine as an antioxidant that exerted a protective effect on cerebral ischemia [42]. Also, the usual analgesic dose of morphine prevented lipid peroxidation due to H2O2 exposure in the glioma cell line [43]. In accordance, the previous study has shown that morphine injection has healed gastric stress ulcers through a reduction in myeloperoxidase activity [44]. In the literature, we found no study assessing the effect of topical opium application and its antioxidant effects after burn injury. Therefore, it can be interpreted that the positive or negative effects of opium on burn healing cannot be due to improving the balance of the oxidant-antioxidant system. Furthermore, despite the altered MDA levels and antioxidant effect in high-dose opium ointment, we found delayed wound healing. So, other mechanistic pathways may be contributed. This point highlights the need for further investigation. In our study, TNF-α levels did not show significant differences between the treatment groups (p>0.05), suggesting that the inflammatory response may not have been significantly modulated by the treatments administered. Conversely, we observed significantly higher serum concentrations of interleukin-10 in the mixed group compared to the SSD group on day 10. The previous study have been shown serum level of IL-10 significantly higher than in control compare with opium addiction subjects [45]. Another study showed that serum IL-10 and TNF-α 48 h after surgery did not differ between control and opium addiction rats [31]. This study investigated the role of early plasma TNF-α and IL-10 as prognostic factors of healing after burn injuries. Keratinocytes produce lL-10 after injury and it is a crucial anti-inflammatory cytokine known to regulate TNF-α signaling negatively [46]. IL-10 facilitates extracellular matrix deposition in the early phases of wound healing. It acts as an anti-scarring therapeutic agent [47]. Proinflammatory biomarkers, including tumor necrosis factor-α (TNF-α) may be useful for monitoring post-burn inflammation and/or immune dysfunction [48], and Thus, it is imaginable that the evaluation these cytokines may serve as a predictive measure of inflammatory homeostasis in burn injuries.

## Limitations

The experimental design of this study can limit the generalizability of the results to humans due to anatomical and physiological differences. We acknowledge that the absence of a naive group limits our ability to compare the effects of treatment to no treatment directly. Including a naive group in future studies to further enhance the validity and generalizability of findings is appreciated. A larger sample size in the 21-day subgroup could provide more comprehensive data and insights into the long-term effects of the treatments. However, the fewer animals in the 21-day subgroup were due to ethical considerations and practical limitations. To address this limitation, we focused on macroscopic appearance to compare the burns over a longer duration. This focus allowed us to evaluate the efficacy of different treatments in managing burn wounds over time without compromising the welfare of the animals involved in the study. In addition, the comb burn model used in this study cannot adequately represent burn in humans. Therefore, further research with appropriate techniques and determining the molecular expression of inflammatory and oxidative stress biomarkers are needed to support the present results. Another limitation is the lack of mechanical testing to establish the tensile strength of the healing tissue in the experimental groups. Inclusion of this method would complement our hypothesis and provide deeper insight into the integrity and functionality of the tissue. In addition, cytotoxic investigation on cell lines, immunohistochemical analysis and molecular evaluation were not conducted; thus, the therapeutic implications of our results cannot be explored satisfactorily. Further studies in mechanical testing, cytotoxicity and immunohistochemical evaluations should be developed in order to build more reliable results, and their scope of study should be extended to diverse cell lines, in vivo models, and more extensive follow-up in order to enhance validity and robustness of the findings. In addition our finding suggests the need for further investigation into the optimal dosing of opium ointments and effects of other natural opioids in various wound healing contexts.

## Conclusion

In conclusion, the results of the study showed that treatment with low doses of topical opium ointment, particularly at concentrations of 0.05% and 0.5%, and in combination with silver sulfadiazine, improved the healing process of experimentally induced burn injuries. Opium-based ointments led to a significant reduction in wound area and lower serum levels of MDA compared to the SSD group. Furthermore, histopathological evaluations showed that the wound-healing activity of opium at the lowest concentration (0.05%) was superior to SSD, and its combination with SSD increased the SSD burn wound repair potential. These findings suggest that opium may have a protective role in thermal-induced injury, and further investigations are needed to elucidate the potential action mechanism of opium. The use of opium-based ointments may offer a promising therapeutic option for burn patients, and our study contributes to the growing body of research on the use of natural compounds for wound healing.
