# Correction: Non-analgesic effects of opioids: Topical application of Eucerin-based ointment containing opium on the healing process of thermal skin damage in rats

**DOI:** 10.1371/journal.pone.0330173

**Published:** 2025-08-11

**Authors:** Omid Mehrpour, Khadijeh Farrokhfall, Kobra Naseri, Samaneh Nakhaee

The Acknowledgement section for this article is missing. The Acknowledgement section is as follows: We want to express our sincere gratitude to Dr. Mehran Hosseini for his valuable collaboration and assistance with the lab work and equipment supply.
